# Oral administration of γ-glutamylcysteine increases intracellular glutathione levels above homeostasis in a randomised human trial pilot study^☆^

**DOI:** 10.1016/j.redox.2017.01.014

**Published:** 2017-01-22

**Authors:** Martin Hani Zarka, Wallace John Bridge

**Affiliations:** School of Biotechnology and Biomolecular Sciences, Faculty of Science, University of New South Wales, Sydney, New South Wales 2052, Australia

**Keywords:** FACS, Fluorescence activated cell sorting, γ-GC, gamma-glutamylcysteine, GCL, glutamate cysteine ligase holoenzyme, GCLC, catalytic subunit of glutamate cysteine ligase, GCLM, modifier subunit of glutamate cysteine ligase, GR, glutathione reductase, γ-GT, gamma-glutamyltransferase, GS, glutathione synthetase, GSH, reduced glutathione, NAC, N-acetylcysteine, NADP, nicotinamide adenine dinucleotide phosphate, PBMC, peripheral blood mononuclear cell x̄ - mean, s, standard deviation, C_0_, initial basal concentration (GSH), C_max_, maximum concentration (GSH), t_max_, time to reach C_max_, AUC, Area under the curve. Overall exposure (to GSH change), Glutathione, Glutamylcysteine, Clinical trial, Homeostasis

## Abstract

**Objective:**

To determine if orally dosed γ-glutamylcysteine (γ-GC) can increase cellular glutathione (GSH) levels above homeostasis. Many chronic and age-related disorders are associated with down-regulation, or impairment, of glutamate cysteine ligase (GCL). This suggests that γ-GC supply may become limiting for the maintenance of cellular GSH at the normal levels required to effectively protect against oxidative stress and any resulting physiological damage.

**Methods:**

GSH levels were measured in lymphocytes of healthy, non-fasting participants before and after single oral doses (2 and 4 g) of γ-GC. Blood samples were immediately processed using high speed fluorescence-activated cell sorting to isolate 10^6^ lymphocytes that were then assayed for GSH content.

**Results:**

A single 2 g dose of γ-GC increased lymphocyte GSH content above basal levels (53±47%, p<0.01, n=14) within 90 min of administration. A randomized dosage (2 and 4 g γ-GC) crossover design was used to explore the pharmacokinetics of this GSH increase. In general, for both dose levels (n=9), GSH increased from initial basal levels over 3 h (t_max_) before reaching maximum GSH concentrations (C_max_) that were near two (2 g γ-GC) to three (4 g γ-GC) fold basal levels (0.4 nmol/10^6^ lymphocytes). Beyond t_max_, GSH levels progressively declined reaching near basal levels by 5 h. The GSH half-life was between 2 and 3 h with exposure (AUC) to increased GSH levels of 0.7 (2 g γ-GC) and 1.8 (4 g γ-GC) nmol.h/10^6^ lymphocytes.

**Conclusions:**

Oral γ-GC is a non-toxic form of cysteine that can be directly taken up by cells and transiently increase lymphocyte GSH above homeostatic levels. Our findings that γ-GC can increase GSH levels in healthy subjects suggests that it may have potential as an adjunct for treating diseases associated with chronic GSH depletion. This trial was registered at anzctr.org.au as ACTRN12612000952842.

## Graphical abstract

**Figure:**
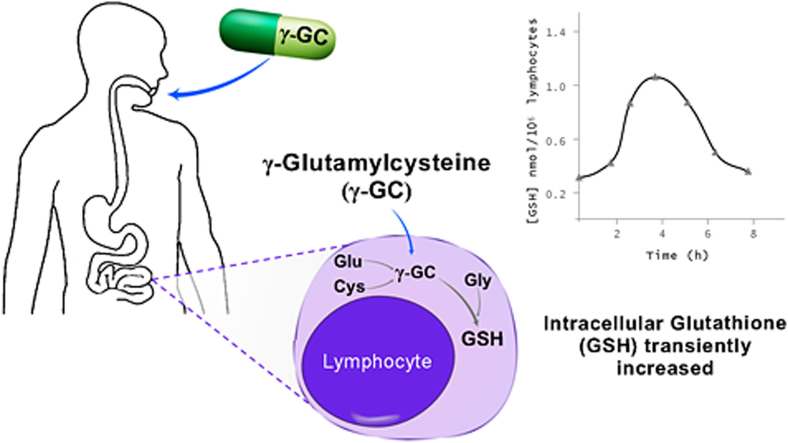

## 1 Introduction

All living organisms have evolved elaborate redox codes that manage metabolic organization and function. These codes dictate and modify the redox chemistry within different cell types, and cellular compartments, during different stages of life cycle and in response to external environmental influences. Disruptions of the redox code that lead to dysregulation of redox steady states will detrimentally impact on cell biochemistry, tissue function and overall health [1]. Glutathione is the most abundant low molecular weight thiol found in cells and plays an important role in the maintenance and regulation of the thiol-redox status of the cell. Thus, the homeostasis of glutathione at optimal concentrations and reduced/oxidised ratios in cell compartments could be argued as being fundamental to a healthy cellular redox [2].

Reduced glutathione (GSH) is a tripeptide (γ-L-glutamyl-L-cysteinylglycine). It is often referred to as the “master antioxidant” and it is produced in the cytosol of all cell types at concentrations up to 10 mM [3]. Beyond its roles as a reducing agent and major antioxidant, GSH is also involved in numerous physiological functions. These include cell cycle regulation, proliferation, apoptosis, xenobiotic metabolism and thiol disulphide exchange. It also serves as a reservoir of cysteine [4].

The intracellular GSH concentration, or homeostasis, is determined by a dynamic balance of synthesis, consumption and transport, and in some tissues; the oxidant levels [4], [5]. Cytosolic GSH *de novo* synthesis occurs in all mammalian cells by two sequential ATP dependent enzyme catalysed reactions. In the first, glutamate cysteine ligase (GCL) forms the unusual γ-peptide bond between L-glutamic acid and L-cysteine to produce γ-glutamylcysteine (γ-GC). In the second, glutathione synthetase (GS) then adds glycine to γ-GC to generate GSH [6], [7]. Cellular GSH homeostasis is controlled by non-allosteric feedback inhibition exerted by GSH on the activity of GCL. There is no such inhibition on GS activity [7].

Indisputable cause and effect links have been demonstrated between changes in GSH levels and/or redox state and chronic diseases, such as Alzheimer's and Parkinson's diseases, diabetes, cystic fibrosis, HIV/AIDS and aging [8], [9], [10], [11], [12], [13]. Though it has been observed in healthy epithelial tissues such as the lung, that GSH expression can increase in response to elevated exposure to oxidants [4], [5], it is generally accepted that, as we age, our body's capacity to maintain an appropriate homeostatically controlled GSH level progressively declines, leaving us vulnerable to many age-related diseases and disorders [2], [14], [15]. A deficiency in GSH manifests itself largely through an increase in susceptibility to oxidative stress. The resulting damage is thought to be a determinant of the onset and progression of many chronic disease states [2]. It is widely thought that drugs or supplements able to elevate glutathione (GSH) levels could have therapeutic potential in treating chronic and age related disorders [8], [9], [10].

Disease associated cellular GSH depletion is often a result of down regulation of expression or lowering of the specific activities of the first biosynthetic enzyme GCL [16]. Rodent studies have shown GCL levels declining with increasing age, corresponding to a lowering of homeostatic GSH levels [17], [18]. The second enzyme involved in GSH synthesis, glutathione synthetase (GS), is a much simpler homodimer composed of two identical catalytic subunits. It generally has a higher specific activity than GCL so that cellular levels of γ-GC are negligible [19]. Many diseases [20], [21], [22], [23] have associated impaired GCL activities from genetic or environmental factors that lower GSH homeostasis to levels that may be insufficient to protect against the onset of oxidative stress. This supports the theoretical potential for the use of γ-GC, the immediate precursor to GSH, as a means to increase cellular GSH levels. As cytosolic concentrations of γ-GC are in the order of 7 μM [19], any passive flow of exogenous γ-GC, unlike GSH, would be directed into the cell [24]. Should cellular GSH depletion arise as a result of damaged regulatory control of GCL activity, NAC or other cysteine prodrugs would theoretically not be expected to be effective in elevating GSH levels above the lowered homeostasis [25]. On the other hand, exogenous γ-GC taken up intact, should feed directly into the unregulated GS enzyme and potentially increase GSH levels above homeostatic levels [26].

Early rodent studies demonstrated in mice that intraperitoneal administered γ-GC could restore depleted GSH content within organs [6]. More recently, γ-GC has been demonstrated to ameliorate oxidative injury in neurons and astrocytes *in vitro* and increases brain glutathione *in vivo* [27]. Further *in vivo* studies of neural [28] cardiac [29] and liver [30] tissues have demonstrated that the extracellular addition of γ-GC ethyl ester also increases intracellular GSH concentrations. [31]. *In vitro* studies with isolated mitochondria have shown that γ-GC can directly replace the role of GSH [32]. The same researchers also determined that γ-GC can take over the antioxidant and neuroprotective functions of GSH by acting as glutathione peroxidase-1 cofactor in a mouse model [33].

In this current human study, we investigated the potential systemic bioavailability of orally administered γ-GC. Dipeptides are not expected to be particularly useful as oral therapeutics as they are often readily hydrolysed by digestive or serum proteases; however, the unusual γ-glutamyl bond found in γ-GC is resistant to hydrolysis by most proteases and aminoproteases [34], [35].

Animal safety trials have demonstrated γ-GC to be safe at limit acute and repeated doses [36]. Change in GSH content of lymphocytes was chosen as a surrogate measure for cellular γ-GC uptake, and hence its bioavailability. Changes in GSH levels in erythrocytes were not determined as, unlike lymphocytes, their metabolism, physiology and structure are not considered representative of most cell types in the body. In spite of having an efficient productive capacity for GSH *de novo* synthesis, they lack a nucleus and most organelles, and are unable to utilize extracellular GSH due to the absence of the membrane bound ectoenzyme γ-glutamyltranspeptidase (γ-GT) [37], [38], [39]. In addition, during the GSH analysis procedure, samples are normally acidified to prevent GSH autoxidation and its hydrolysis by γ-GT. This, however, is ineffective for erythrocytes which release a large amount of iron that reacts with GSH even under acidic conditions [40], [41]. Similarly, the monitoring of any changes in plasma GSH or γ-GC levels was not considered relevant to the study objectives since any increase in plasma GSH would require cellular uptake of exogenous γ-GC followed by secretion of synthesised GSH into the plasma. A single oral dose of γ-GC, rather than multiple doses over a period of weeks or months, was investigated. If γ-GC can effectively increase GSH above homeostasis, then the effect should be able to be observed after a single dose.

## 2 Materials and methods

### 2.1 Study protocol

The study's focus was to determine whether single doses (2 and 4 g) of orally ingested γ-GC can transiently increase the GSH content of lymphocytes. The study was sponsored by the University of New South Wales (UNSW) and approved by the UNSW Human Research Ethics Committee (HREC Ref#HC12511). It was prospectively registered with the Australian New Zealand Clinical Trials Registry (Trial ID ACTRN12612000952842) and filed under the Clinical Trial Notification (CTN) scheme of the Therapeutics Goods Administration (TGA, Australia; Ref #20120750). The trial was conducted at the UNSW according to the principles of the Declaration of Helsinki. Thirteen healthy adult male and female volunteer subjects (aged 25–70) were recruited by word of mouth from friends, family and work colleagues The γ-GC (CAS No. 636-58-8) administered in the study was provided by Biospecialties International, Mayfield, NSW, Australia as a sodium salt (Glyteine®). The γ-GC and placebo (glucose) were both packaged in identical 500 mg capsules.

A non-fasting requirement for participants was included to eliminate the risk of substrate (in particular cysteine) limitation decreasing GSH levels below homeostasis and thereby confounding the experimental interpretation. If, following overnight fasting, a subject's lymphocyte GSH levels were below homeostasis due to substrate limitation, any observation of increased GSH levels following the γ-GC administration would have been potentially due to a return to homeostasis rather than an increase above homeostasis. No alcohol consumption was allowed on the day of the trial due to its potential to deplete cellular GSH [42]. Throughout the day, subjects were requested to record all food and drink consumption.

The study involved a single dose of 2 g γ-GC (4×500 mg capsules) plus 2 g placebo (4×500 mg capsules) or 4 g γ-GC (8×500 mg capsules) taken with water. For the *before and after* study, subjects (n=13) were administered only the 2 g γ-GC dose, with blood samples being taken before γ-GC administration and at 90 min afterwards. The pharmacokinetic component of the study aimed to investigate the rates of increase in lymphocyte GSH content and the expected subsequent decline to basal homeostatic levels. Six subjects were selected for a randomized double blind crossover 2 and 4 g dose comparison (see Table 1 for subject demographics). Blood samples were taken over periods of up to 7 h after γ-GC administration. The subjects were tested with the opposite dose after a minimum two week washout period, with the dose sequences for individuals selected by block randomization. The dose selection process, coding and capsule management was performed by a third party not associated with the trial objectives, leaving both the investigators and subjects blinded to the γ-GC dose being administered.

### 2.2 Blood processing

Initial attempts at isolating lymphocytes from blood using Ficoll-Hypaque density gradient centrifugation resulted in unacceptably high variations in the GSH analysis. When examined microscopically, variable levels of erythrocyte contamination were observed. This issue was overcome by using high throughput fluorescence activated cell sorting FACS, which allows the collection of 10^6^ lymphocytes in less than 5 min. This eliminated sample contamination by erythrocytes and provided sufficient lymphocytes to detect and measure GSH using a conventional microtitre plate method.

All reagents and equipment used in the study were sourced from BD Australia (North Ryde, NSW). Blood samples (5 ml) were taken via syringe or cannula into anticoagulant (heparin) treated tubes. The samples were immediately processed to isolate the lymphocytes and stabilise their GSH content. An ammonium chloride-based lysing solution protocol was used to clear the erythrocytes. Briefly, whole blood (2 ml) was dispensed into Falcon tubes containing BD Pharm Lyse™ solution (20 ml, ×1), mixed by inversion, and incubated at room temperature for 15 min before centrifugation at 200×*g* for 5 min. The supernatant was aspirated and the pellet resuspended in PBS (2 ml) containing heat-inactivated foetal bovine serum (1%). FACS was then used to rapidly obtain a highly concentrated and purified sample of lymphocytes. Using a BD Influx™ cell sorter set-up for high through-put (approximately 30,000 particles per second) lymphocytes were gated using light-scattering characteristics and 1×10^6^ lymphocytes were collected in Eppendorf tubes containing PBS (200 µl). The lymphocyte gate was validated on repeated occasions by using the labelled monoclonal antibody staining to CD45 method [43].

At the end of each FACS run, the tubes were immediately made up to 1 ml with HCl (100 mM) solution containing 0.1% Triton X100. The resulting lysates were either assayed immediately or stored at −20 °C for up to 2 days before GSH analysis. GSH concentrations were determined using a modified GR enzyme recycling protocol [44]. Briefly, in 96 well microtitre plates, aliquots of the lysates (100 µl) along with GSH standards (100 µl, 0.1–5 µM) were added in duplicate to wells containing phosphate buffer (40 µl, 143 mM, pH 7.4) solution comprising glucose-6-phosphate (1.7 mM), DNTB [5,5′-dithiobis(2-nitrobenzoic acid, (4.9 mM)], NADP+(0.17 mM), glucose-6-phosphate dehydrogenase (5 IU/ml), and EDTA-Na (6.3 mM). To generate NADPH, the plate was incubated for 10 min at room temperature after which GR (20 µl, 10 IU/ml PBS) was added rapidly with a 12-channel pipette. The plate was incubated in a plate reader (BMG FLUOstar OPTIMA) at room temperature and read at 412 nm at 60 s intervals for 10 min. The GSH concentrations were determined from generated standard curves and were expressed as GSH nmol/10^6^ cells.

### 2.3 Statistics and pharmacokinetic analysis

A students two tailed paired *t*-test was used to determine the significance (*p*) of differences in basal (pre-dose) and after γ-GC dose lymphocyte GSH levels. For the pharmacokinetic GSH data, the t_max_ and C_max_ values were estimated graphically using Excel. The Area Under the Curve (AUC) values were calculated using the linear trapezoidal rule [45] AUC=Σ_0-last_ ½ (C_1_+C_2_)(t_2_−t_1_), which was modified to ΔAUC=Σ_0-last_ ½ (C_1_+C_2_−2C_0_)(t_2_−t_1_) to gain a measure of γ-GC's direct distribution effect on lymphocyte GSH levels.

## 3 Results

No adverse effects were reported by any of the study participants on the days of administration and during the subsequent 7 days. During the performance of the pharmacokinetic trial, one of the subjects withdrew from the study due to discomfort with the blood sampling procedure.

**Fig. 1:**
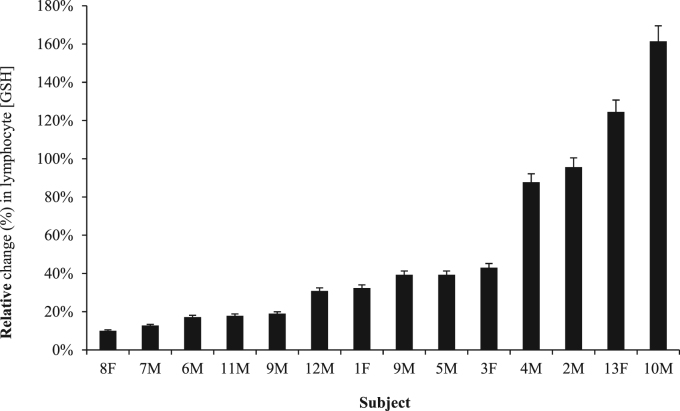
The relative percent increase above basal glutathione (GSH) content in the lymphocytes of human healthy non-fasting adults (n=14) 90 min after the oral administration of 2 g γ-glutamylcysteine (γ-GC). The average GSH increase was 53±47% (p<0.01).

For the initial 2 g γ-GC trial involving GSH measurements before and 90 min post administration, the lymphocyte GSH content increased in all 14 trials (n=13 subjects) by an average of 53±47% (standard deviation, s; p<0.01) with a range of 10–161% (Fig. 1). The average post dose GSH content was 0.41±0.14 nmol/10^6^ cells relative to an initial basal level of 0.29±0.12 nmol/10^6^ cells (Supplemental Table 1).

**Fig. 2:**
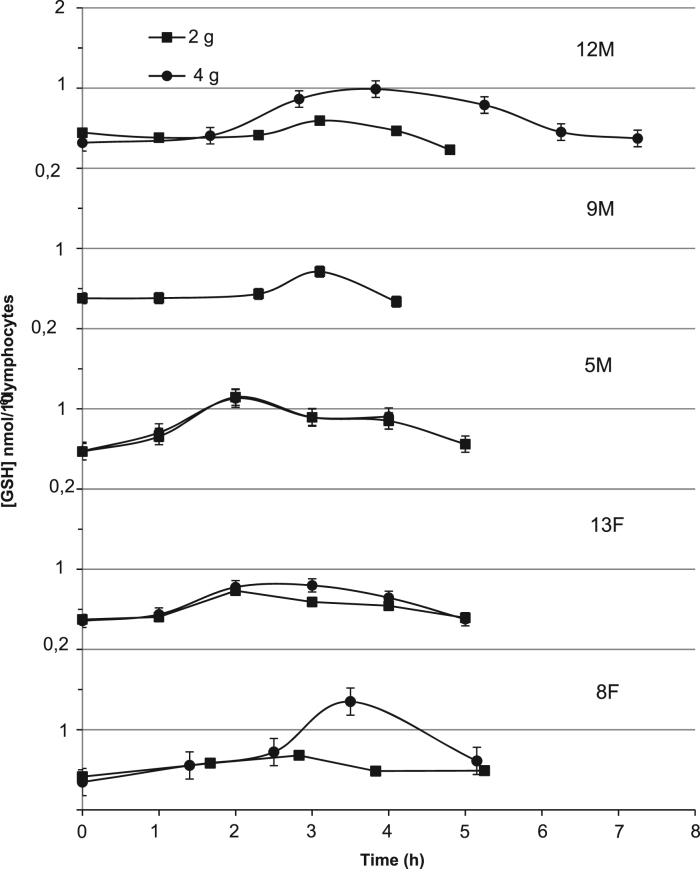
Changes in glutathione (GSH) content of lymphocytes in individual human healthy non-fasting adults (n=5) following the randomized crossover oral administration of 2 and 4 g γ-glutamylcysteine (γ-GC) with a minimum two weeks separation between doses.

Data from the 9 M 4 g γ-GC pharmacokinetic study was excluded due to compromised GSH analysis. In general, the lymphocyte GSH content for all five subjects tended to increase following γ-GC administration over a period of 2–4 before declining by 4–7 h to near original basal levels (Fig. 2). The average maximum increase in GSH level relative to initial basal levels was approximately 3 fold in the 4 g γ-GC dose and 2 fold in the 2 g, with the average half-life of increases in GSH across both doses being 2.4±0.4 h. The average exposure (ΔAUC) to increased GSH levels was 2.5 fold higher for 4 g dose than that observed for the 2 g, though increased trial numbers would be required to confirm the significance of any of these observed dose dependent differences, (Table 2, Fig. 2).

## 4 Discussion

The objective of this study was to determine whether orally administered γ-GC has the potential to systemically increase cellular GSH levels, which if confirmed would identify γ-GC as a therapeutic candidate for the broad range of clinical conditions associated with GSH depletion. In all 23 trials undertaken in this study, the administration of γ-GC resulted in a significant increase in lymphocyte GSH content. For the pharmacokinetic trials, following γ-GC administration, there was a steady increase in lymphocyte GSH levels as, presumably, the γ-GC is progressively absorbed from the gastrointestinal tract via the hepatic portal system and distributed throughout the body via the vascular system, where it is taken up intact by lymphocytes and putatively other cell types. Once inside the cells the γ-GC is converted into GSH by GS activity. The rapid increase in GSH to C_max_ levels over 2–3 h suggests that the release rate (K_r_) of γ-GC from the capsules was not a rate limiting step. The C_max_ GSH levels were 2–3 fold higher than basal (C_0_), which resulted in relatively high transient exposure levels (ΔAUC). This will need to be addressed when considering therapeutic applications and will likely require the use of drug delivery systems that can best control and sustain a desired K_r_ from oral γ-GC formulations. The decline in GSH levels towards homeostasis, over the 2–3 h post t_max_, was likely due to the slowing of cellular γ-GC synthesis arising from elevated GSH levels exerting feedback inhibition on the GCL activity [7]. The mechanism by which γ-GC is taken up by cells intact is yet to be determined, although the existence of a specific transporter has previously been postulated [46]. The observed variation in absolute GSH response between individual subjects following γ-GC dosage could be expected with the 63–110 kg range of subject body weights (Table 1).

In this study, the basal GSH concentrations in lymphocytes were estimated to be in the order of 0.3–0.4 nmol/10^6^ cells. Compared to the variability of GSH concentrations reported in the literature for erythrocytes and plasma, lymphocyte GSH concentrations are surprisingly consistent in the order of 0.6 nmol/10^6^ cells. Table 3 collates reported healthy lymphocyte GSH levels (controls) from a range of studies investigating the relationship between GSH depletion and diseases.

It has been well established that the lymphocytes of HIV/AIDS patients have low GSH content [47], [48]. To address this, several human clinical trials have investigated the potential of NAC to replenish GSH. All have failed to give sufficient benefit to gain regulatory approval [49]. In a trial involving a dosage of 30 mg/kg body weight (bw) (2 g for a 70 kg person) of NAC, no change after 2 and 4 h was observed in the peripheral blood mononuclear cells (PBMC) of the healthy control group (n=6) and only moderate increases in the HIV positive group (n=9) [50]. Another study investigated a daily dose of 1.8 g of NAC for 2 weeks in six HIV positive patients. Again, no significant increase in PBMC GSH was observed. It was concluded that this failure to increase GSH levels was related to inadequate rates of GSH synthesis in HIV patients rather than due to increased GSH consumption arising from any increased exposure to oxidants [51]. A further study used a daily dosage of 2×200 mg NAC for 3 days which resulted in a significant decrease in lymphocyte GSH in healthy adults (n=46) [52]. The findings from our study suggest that γ-GC may have therapeutic potential in the treatment of HIV positive patients.

The failure of NAC in these studies is unsurprising. Even though NAC has been proven to be invaluable in cases of acute GSH depletion, such as in the case of acetaminophen (APAP) overdose [53], its benefit in cases of chronic depletion of GSH has proven to be limited [54], [55], [56]. This is likely due the differences in the cause of GSH depletion. In the case of APAP overdose, detoxification by conjugation with GSH and the reactive intermediate, outstrips the production of GSH resulting in oxidative stress [53]. As such, the supply of substrates for GSH biosynthesis, in this case NAC, is an effective treatment. In chronic diseases a progressive lowering of the homeostatic control level of GSH is more likely the effect of diminished capacity to biosynthesize GSH due to damage or down regulation of the GCL enzyme. In support of this, cysteine is not an essential amino acid and is unlikely to be in limited supply as modern diets are rich in cysteine [57] and in methionine, which can be readily converted to cysteine [58]. It is hardly surprising then that the treatment of chronic GSH depletion by supplementation with cysteine prodrugs, such as NAC, is ineffective. On the other hand, GGC gives clinicians a tool to increase GSH levels in chronically ill patients by bypassing the GCL enzyme.

In conclusion, we postulate that exogenous γ-GC is a non-toxic form of cysteine that can be directly taken up by cells and converted to GSH by GS activity. Our findings that γ-GC can increase GSH levels in healthy subjects suggests that it may have potential as an adjunct for treating diseases associated with chronic GSH depletion arising from down regulated or damaged regulatory control of GSH homeostasis.

## Sources of support

The clinical trial described in the report was sponsored by the University of New South Wales.

## Figures and Tables

**Table 1:** Subject demographics.

	*before and after* trial (n=13)	Pharmacokinetic trial (n=6)
Age (years)	46±15	55±12
Male (n)	9	3
Female (n)	4	2
Regular smokers (n)	0	0
Race	12 Caucasian	All Caucasian
	1 Eurasian (50:50)	
Male weight (kg)	90±14	88±20
Female weight (kg)	69±7	65±2
Male height (cm)	179± 5	180±7
Female height (cm)	167±9	160

**Table 2:** Pharmacokinetic parameters for lymphocyte glutathione (GSH) levels following the oral administration of 2 or 4 g γ-glutamylcysteine (γ-GC).

Subject	C_0_	2 g γ-GC					
		C_max_	C_max_/C_0_	t_max_	AUC	ΔAUC	Half-life (h)
				(h)			
12M	0.443	0.597	1.4	3.1	2.11	0.05	2.1
9M	0.379	0.710	1.9	3.1	1.89	0.36	2.5
5M	0.467	1.175	2.5	2.3	4.06	1.72	2.5
13F	0.374	0.740	2.0	2.1	2.66	0.79	2.5
8F	0.413	0.685	1.7	2.8	2.83	0.67	1.9
x̄		0.781	1.9	2.7	2.71	0.72	2.3
s		0.226	0.4	0.5	0.85	0.63	0.3
		4 g γ-GC					
12M	0.317	0.992	3.1	3.7	4.56	2.26	3.2
5M	0.473	1.140	2.4	2.2	3.42	1.52	2.5
13F	0.356	0.822	2.3	2.5	3.02	1.24	2.5
8F	0.347	1.350	3.9	3.5	3.98	2.20	2.6
x̄	0.397	1.076	2.9	3.0	3.75	1.81	2.6
s	0.055	0.224	0.7	0.7	0.67	0.50	0.5

C_max_ – Maximum [GSH]. [GSH] units - nmol GSH]/10^6^ lymphocytes. C_0_ – Basal [GSH] before GSH administration. T_max_ – Time (h) to reach C_max_. AUC – Area under the curve (nmol GSH h/10^6^ lymphocytes) based on C_t_ values. ΔAUC – based on (C_t –_ C_0_).

**Table 3:** Reported human glutathione content in healthy human lymphocytes.

Average [GSH] nmol/10^6^ cells^a^	Subjects	Reference
	(n)	
0.855	5	[59]
0.740	10	[60]
0.537	41	[61]
0.447	41	
0.307	42	
0.672	105	[62]
0.702	93	[63]
0.687	61	
0.597	86	
0.562	48	[64]

a Data that was originally reported as [GSH]/mg cellular protein have been converted to nmol GSH/10^6^ cells on the basis of an average protein concentration of 0.025 mg/10^6^ cells [63].
