# Supplementary material for: Oral administration of γ-glutamylcysteine increases intracellular glutathione levels above homeostasis in a randomised human trial pilot study
Source: Redox Biol. 2017 Jan 22;11:631–6. doi: 10.1016/j.redox.2017.01.014 (PMC5284489; doi:10.1016/j.redox.2017.01.014)
Supplement: Supplementary material [file mmc1.docx]

Supplemental Table 1. The effect of oral γ-glutamylcysteine (γ-GC) administration (2 g) on lymphocyte glutathione (GSH) levels after 90 minutes in healthy non-fasting human subjects.

| Subject*/γ-GC dose (g) | Experimental replicate No. | [GSH] (nmole/10^6^ lymphocytes) | | | Percent change in GSH |
| --- | --- | --- | --- | --- | --- |
|  |  | Basal (0 min) | Post dose (90 min) | Change |  |
| 1F | 1 | 0.278 | 0.368 | 0.090 | 32% |
| 2M | 1 | 0.160 | 0.313 | 0.153 | 96% |
| 3F | 1 | 0.178 | 0.255 | 0.077 | 43% |
| 4Mg | 1 | 0.204 | 0.383 | 0.179 | 88% |
| 5M | 1 | 0.356 | 0.496 | 0.140 | 39% |
| 6M | 1 | 0.383 | 0.449 | 0.066 | 17% |
| 7M | 1 | 0.282 | 0.318 | 0.036 | 13% |
| 8F | 1 | 0.271 | 0.299 | 0.028 | 10% |
| 9M | 1 | 0.297 | 0.353 | 0.056 | 19% |
| 9M | 2 | 0.286 | 0.437 | 0.151 | 53% |
| 10M | 1 | 0.236 | 0.617 | 0.381 | 161% |
| 11M | 1 | 0.352 | 0.415 | 0.063 | 18% |
| 12M | 1 | 0.599 | 0.784 | 0.184 | 31% |
| 13F | 1 | 0.143 | 0.321 | 0.178 | 124% |
|  | | n | x̄ | s | %RSD |
| Basal [GSH] - all trials | | 14 | 0.29 | 0.12 | 40% |
| Basal [GSH] - all subjects | | 13 | 0.29 | 0.12 | 42% |
| [GSH] at 90 min | | 14 | 0.41 | 0.14 | 34% |
| Percent change in [GSH] | | 14 | 53% | 47% | 88% |
| p (basal vs post dose) | | 14 | 0.00017 |  |  |

n = number of tests; x̄ = average; s = standard deviation; %RSD = percent relative standard deviation; R – replicate trial. # Statistical significance (*p*) between [GSH] for basal and 90 min post dose was calculated using Students T test, two tailed, paired analysis. Average analytical percent error (s/x̄) between assay replicates was 12.4%. *Subject coding: (F – female; M – Male), (1 = youngest; 13 = oldest).
